# Comparison of Plasma and Urine Biomarker Performance in Acute Kidney Injury

**DOI:** 10.1371/journal.pone.0145042

**Published:** 2015-12-15

**Authors:** Gunnar Schley, Carmen Köberle, Ekaterina Manuilova, Sandra Rutz, Christian Forster, Michael Weyand, Ivan Formentini, Rosemarie Kientsch-Engel, Kai-Uwe Eckardt, Carsten Willam

**Affiliations:** 1 Department of Nephrology and Hypertension, Friedrich-Alexander-University Erlangen-Nürnberg, Erlangen, Germany; 2 Biomarker Assessments, Roche Diagnostics GmbH, Penzberg, Germany; 3 Department of Cardiac Surgery, Friedrich-Alexander-University Erlangen-Nürnberg, Erlangen, Germany; 4 Biomarker & Experimental Medicine, F. Hoffmann-La Roche Ltd, Basel, Switzerland; University of Florida, UNITED STATES

## Abstract

**Background:**

New renal biomarkers measured in urine promise to increase specificity for risk stratification and early diagnosis of acute kidney injury (AKI) but concomitantly may be altered by urine concentration effects and chronic renal insufficiency. This study therefore directly compared the performance of AKI biomarkers in urine and plasma.

**Methods:**

This single-center, prospective cohort study included 110 unselected adults undergoing cardiac surgery with cardiopulmonary bypass between 2009 and 2010. Plasma and/or urine concentrations of creatinine, cystatin C, neutrophil gelatinase-associated lipocalin (NGAL), liver fatty acid-binding protein (L-FABP), kidney injury molecule 1 (KIM1), and albumin as well as 15 additional biomarkers in plasma and urine were measured during the perioperative period. The primary outcome was AKI defined by AKIN serum creatinine criteria within 72 hours after surgery.

**Results:**

Biomarkers in plasma showed markedly better discriminative performance for preoperative risk stratification and early postoperative (within 24h after surgery) detection of AKI than urine biomarkers. Discriminative power of urine biomarkers improved when concentrations were normalized to urinary creatinine, but urine biomarkers had still lower AUC values than plasma biomarkers. Best diagnostic performance 4h after surgery had plasma NGAL (AUC 0.83), cystatin C (0.76), MIG (0.74), and L-FAPB (0.73). Combinations of multiple biomarkers did not improve their diagnostic power. Preoperative clinical scoring systems (EuroSCORE and Cleveland Clinic Foundation Score) predicted the risk for AKI (AUC 0.76 and 0.71) and were not inferior to biomarkers. Preexisting chronic kidney disease limited the diagnostic performance of both plasma and urine biomarkers.

**Conclusions:**

In our cohort plasma biomarkers had higher discriminative power for risk stratification and early diagnosis of AKI than urine biomarkers. For preoperative risk stratification of AKI clinical models showed similar discriminative performance to biomarkers. The discriminative performance of both plasma and urine biomarkers was reduced by preexisting chronic kidney disease.

## Introduction

Novel renal biomarkers which have been identified by genomic, proteomic and metabolomic methods have the potential to detect minor injury of glomeruli and tubules in response to various insults even when creatinine levels are not yet elevated [1]. Consequently, biomarkers have been intensively investigated in the last years and have been shown to be potentially suitable for risk stratification and early diagnosis of acute kidney injury (AKI), prediction of AKI progression, severity, and outcomes or need for renal replacement therapy (RRT) [2–4]. Most promising candidates are neutrophil gelatinase-associated lipocalin (NGAL), kidney injury molecule 1 (KIM1), liver fatty acid-binding protein (L-FABP), cystatin C, interleukin-18 (IL-18), and the combination of two cell cycle arrest markers tissue inhibitor of metalloproteinase-2 (TIMP-2) and insulin-like growth factor binding protein 7 (IGFBP7) [5–10]. Renal biomarkers have been assessed in plasma and/or urine. Each sample material has its own advantages and drawbacks. Plasma is obtainable even in anuric patients and is less prone to bacterial contamination. However, changes in serum biomarker concentrations are not necessarily related to decreased kidney function alone and can be the product of a systemic response [11]. In contrast, urine samples can be noninvasively collected, contain a reduced number of interfering proteins, and may have higher specificity for kidney damage irrespective of systemic pathology [12]. However, urine collection can be limited in oliguric patients, and urinary biomarkers may be influenced by changes in renal concentrating mechanisms and urine flow rate due to the patient’s hydration status or the use of diuretics. Moreover, a standard methodology for collection, processing, storage, and measurement of urinary biomarkers is lacking resulting in inconsistent sample handling in clinical studies. In addition, there is controversy about the validity of normalizing urine biomarkers for urinary creatinine [13–16]. So far to our best knowledge a systematic comparison of biomarkers in blood and urine in the same patient cohort has not been available. In this study we therefore prospectively investigated the performance of renal biomarkers in urine and plasma in adult patients developing AKI after cardiac surgery. Based on published data [4,17] we tested if urine biomarkers are superior to plasma biomarkers. In particular we 1) directly compared the performance of creatinine, NGAL, KIM1, L-FABP, cystatin C, and albumin in urine with their plasma counterparts (when applicable), 2) analyzed 15 additional biomarkers in plasma and in urine, 3) evaluated combinations of clinical scores and renal biomarkers for AKI risk stratification, and 4) assessed the impact of preexisting chronic kidney disease (CKD) on biomarker performance.

## Methods

### Patient Population

Adult patients (age 18 years or older) undergoing cardiac surgery using cardiopulmonary bypass (CPB) at the University Hospital Erlangen between July 2009 to August 2010 were prospectively enrolled. The study was approved by the local Institutional Review Board (Ethik-Kommission der Medizinischen Fakultät der Friedrich-Alexander-Universität Erlangen-Nürnberg, #4010), and all participants provided written informed consent before enrollment. Exclusion criteria were preexisting hemodialysis-dependent end stage renal disease, previous kidney transplantation, immunosuppressive medication, and pregnancy. Postoperative AKI within 3 days after surgery was defined by Acute Kidney Injury Network (AKIN) serum creatinine criteria without considering the urine output criteria [18]. Early postoperative AKI based on novel renal biomarkers was defined to occur within the first 24 hours after operation. For each patient plasma and urine samples were obtained on the day before surgery and 2, 4, 24, 48, 72 hours after completion of CPB (which was defined as time point 0) as well as on day 7 after surgery for AKI patients. Preoperative urine samples were collected from spontaneous, mid-stream micturition, postoperative urine samples were directly drawn from urinary catheters. All urine samples were analyzed irrespective of abnormalities (microhematuria or leukocyturia) in dipstick urinalysis and microscopic examination of the urinary sediment. Plasma creatinine values were obtained in routine care for every patient every morning throughout the entire hospital stay. The estimated glomerular filtration rate (eGFR) was calculated using the re-expressed Modification of Diet in Renal Disease (MDRD) study 4-variable equation [19]. CKD (stage ≥2) was determined according to the KDIGO definition based on preoperative and historical outpatient and in-hospital creatinine and albuminuria values [20]. The risk for postoperative AKI was calculated using the European System for Cardiac Operative Risk Evaluation (EuroSCORE) [21] and Cleveland Clinic Foundation (CCF) score [22]. Both EuroSCORE and CCF score were grouped into three risk categories: low (0–2), medium (3–5), and high risk (>5).

In addition, plasma and mid-stream urine samples from 30 self-declared healthy volunteers (mean age 43.3 years, 48.3% male) were collected to assess normal reference ranges for AKI biomarkers. Normal urine albumin/creatinine ratio (ACR) has been reported earlier [20].

### Biomarker Measurement

Urine and plasma samples were kept at 4°C and processed within 30 min after collection. Urine samples were centrifuged at 1500 rpm for 5 min and EDTA-plasma at 3000 rpm for 10 min. The obtained supernatants were stored in aliquots at -80°C for a median of 5 months until assayed. Recent studies showed high stability of urinary biomarkers under these processing conditions [23–25]. Creatinine concentrations in urine and plasma were determined by an enzymatic assay (Creatinine Plus, Roche Diagnostics GmbH, Mannheim, Germany). Plasma and urinary NGAL (Kit 036, BioPorto Diagnostics A/S, Gentofte, Denmark), plasma and urinary L-FABP (Roche Diagnostics GmbH) as well as urinary KIM1 (Duoset, R&D Systems, Minneapolis, MN) were measured via ELISA methods. Urine and plasma cystatin C as well as urine albumin were measured using latex particle-enhanced immunoturbidimetric assays (Tina-quant, Roche Diagnostics GmbH). Multiplex immunoassay analyses in plasma and urine samples were performed using the Human CustomMAP and the Human KidneyMAP^®^ respectively (Rules-Based Medicine, Austin, TX) based on Luminex technology. All laboratory investigators were blinded to each patient’s clinical information, and all measurements were made at least in duplicate.

### Statistical Methods

For summary tables, continuous variables were summarized by mean and standard deviation or median with range, and categorical variables were summarized by counts and percentages. Comparison of distributions for AKI versus non-AKI group was performed with Wilcoxon rank-sum test for continuous variables or Fisher's exact test for categorical variables. P-values below 0.05 were considered significant (two tailed). However, no correction for multiple comparisons was made.

Diagnostic performance for discriminating AKI from non-AKI was assessed using Receiver Operation Characteristic (ROC) curves and corresponding area under the curve (AUC). The confidence intervals for AUCs were calculated with the DeLong method [26].

A multivariable analysis was conducted to analyze combinations of biomarkers and composites of clinical scores and biomarker information. First, logistic regression models were built with AKI as dependent variable; and clinical score and one baseline biomarker log-transformed as independent variable. Predicted probabilities of the regression models were used for assessment of classification performance. To determine whether addition of biomarker testing significantly improved the performance of clinical scores DeLong tests [26] were performed. Second, multivariable analysis was used to select a combination of biomarkers with the best classification performance.

The variable selection was done with the LASSO logistic regression procedure [27]. The classification performance of the corresponding models was assessed via cross-validated AUC. All computations were performed using R version 2.13.1R Development Core Team (2011) (http://www.R-project.org). Lasso logistic regression was done using glmnet 1.7.1 software (http://cran.r-project.org).

## Results

### Patient Characteristics and Renal Function Changes

We analyzed 110 unselected adult patients undergoing cardiac surgery using CPB. Table 1 presents demographic and clinical characteristics of the patient population. Postoperative AKI was defined by AKIN serum creatinine criteria only for a 72h interval following discontinuation of CPB to account for perioperative hemodilution. Within 3 days after surgery 37 (34%) participants developed AKI: 28 (76%) at stage 1, 3 (8%) at stage 2, and 6 (16%) at stage 3. Five patients (5%) received acute RRT, 3 of them had preexisting CKD stage 3 or greater, and 2 (2%) died before discharge. Patients who developed AKI were older, had higher EuroSCORE and CCF score values, more often had preexisting CKD, more frequently underwent redo surgery, valve or combined coronary artery bypass grafting and valve surgery, were longer mechanically ventilated, more likely to be hemodynamically unstable, and to need postoperative intra-aortic balloon pump. AKI patients stayed longer in hospital, intensive and intermediate care units than patients without AKI (S1 Table).

**Table 1:** Characteristics of study participants stratified by AKI.

	All (n = 110)	Non-AKI (n = 73)	AKI 1–3 (n = 37)	P
**Demographics**				
**Age (years), mean (SD)**	69.8 (9.6)	68.2 (10.0)	73.0 (7.7)	*0*.*01*
**Gender, n (%)**				
male	84 (76.4)	58 (69)	26 (31)	0.34
female	26 (23.6)	15 (57.7)	11 (42.3)	
**BMI (kg/m** ^**2**^ **), mean (SD)**	28.3 (4.3)	28.1 (3.8)	28.7 (5.2)	0.63
**EuroSCORE, mean (SD)**	5.5 (2.7)	4.6 (2.4)	7.1 (2.7)	*<0*.*001*
**CCF score, mean (SD)**	3.1 (1.9)	2.6 (1.6)	4.0 (2.1)	*<0*.*001*
**Preoperative renal function**				
**Chronic kidney disease, n (%)**				
no	63 (57.3)	51 (81.0)	12 (19.0)	*<0*.*001*
yes	47 (42.7)	22 (46.8)	25 (53.2)	
**CKD stage, n (%)**				
CKD 2	1 (2.1)	0 (0.0)	1 (100.0)	0.61
CKD 3	42 (89.4)	21 (50.0)	21 (50.0)	
CKD 4	4 (8.5)	1 (25.0)	3 (75.0)	
**AKI in anamnesis, n (%)**				
no	105 (95.5)	71 (67.6)	34 (32.4)	0.33
yes	5 (4.5)	2 (40.0)	3 (60.0)	
**RRT in anamnesis, n (%)**				
No	108 (98.2)	71 (65.7)	37 (34.3)	0.54
yes	2 (1.8)	2 (100.0)	0 (0.0)	
**Preoperative pCreatinine (mg/dl), mean (SD)**	1.21 (0.46)	1.15 (0.41)	1.33 (0.52)	0.06
**Preoperative eGFR, (ml/min per 1.73 m** ^**2**^ **), n (%)**				
≥ 60	60 (54.5)	46 (76.7)	14 (23.3)	*0*.*03*
30 to 59	45 (40.9)	24 (53.3)	21 (46.7)	
< 30	5 (4.5)	3 (60.0)	2 (40.0)	
**Preoperative proteinuria by dipstick, n (%)**				
no	97 (88.2)	65 (67)	32 (33)	0.75
yes	13 (11.8)	8 (61.5)	5 (38.5)	
**Characteristics of surgery**				
**Cardiac catheterization in the last 72 hours, n (%)**				
no	4 (3.7)	3 (75.0)	1 (25.0)	1.00
yes	104 (96.3)	70 (67.3)	34 (32.7)	
**Status of procedure, n (%)**				
elective	68 (61.8)	46 (67.6)	22 (32.4)	0.83
urgent or emergent	42 (38.2)	27 (64.3)	15 (35.7)	
**Incidence, n (%)**				
first	104 (94.5)	72 (69.2)	32 (30.8)	*0*.*02*
redo	6 (5.5)	1 (16.7)	5 (83.3)	
**Surgery, n (%)**				
CABG	70 (63.6)	53 (75.7)	17 (24.3)	*0*.*01*
valve	17 (15.4)	7 (35.7)	10 (64.3)	
CABG and valve	16 (14.5)	7 (43.7)	9 (56.3)	
thoracic aorta	7 (6.4)	6 (85.7)	1 (14.3)	
**Surgery duration (min), median (min-max)**	204 (126–384)	199 (126–368)	211 (139–384)	0.18
**Mechanical ventilation time (h), median (min-max)**	11 (6–533)	10 (6–34)	14 (6–533)	*<0*.*001*
**CPB perfusion time (min), median (min-max)**	78 (36–248)	77 (36–196)	80 (37–248)	0.36
**Aortic cross clamp time (min), median (min-max)**	46 (20–130)	43 (20–138)	52 (20–107)	0.09
**Intraoperative min MAP (mmHg), mean (SD)**	37.6 (8.1)	38.1 (7.5)	36.6 (9.1)	0.33
**Epinephrine use, n (%)**	15 (14)	3 (4)	12 (32)	*<0*.*001*
**Preoperative IABP, n (%)**				
no	107 (97.3)	72 (67.3)	35 (32.7)	0.26
yes	3 (2.7)	1 (33.3)	2 (66.7)	
**Postoperative IABP, n (%)**				
no	100 (90.9)	72 (72.0)	28 (28.0)	*<0*.*001*
yes	10 (9.1)	1 (10.0)	9 (90.0)	
**Intraoperative diuresis (ml), median (min-max)**	870 (120–2250)	860 (120–2250)	900 (120–2050)	0.47

For categorical variables the first column (“All”) shows column percentages. The columns “non-AKI” and “AKI” contain row percentages. Abbreviations: AKI, acute kidney injury; BMI, body mass index; CABG, coronary artery bypass grafting; CCF, Cleveland Clinic Foundation; CKD, chronic kidney disease; CPB, cardiopulmonary bypass; eGFR, estimated glomerular filtration rate; EuroSCORE, European System for Cardiac Operative Risk Evaluation; IABP, intra-aortic balloon pump; MAP mean arterial pressure; max, maximum; min, minimum; RRT, renal replacement therapy; SD, standard deviation.

### Early diagnosis of postoperative AKI with biomarkers in plasma

**Fig 1:**
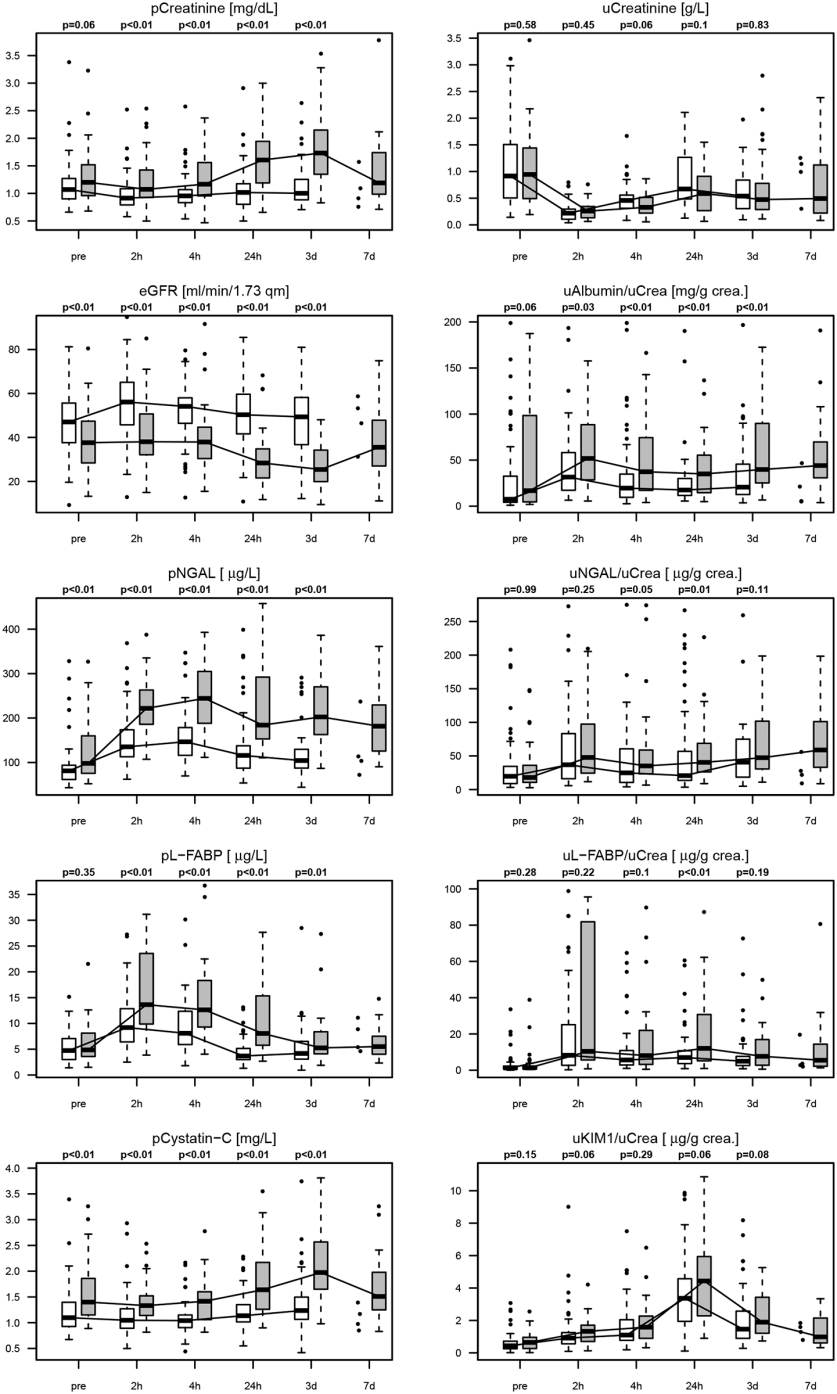
Renal biomarkers in plasma and urine. Time course box blot analysis for plasma (left columns) and urinary (right column) biomarkers including creatinine, eGFR, albumin, NGAL, L-FABP, cystatin C and KIM1. Bright columns (left at each time point) represent non-AKI patients, dark columns (right at each time point) AKI patients. Biomarkers in urine are normalized to urinary creatinine (uCrea). Boxplots display median, 25th and 75th percentiles. The unadjusted p-values of Wilcoxon rank-sum test are depicted above the boxplots.

**Fig 2:**
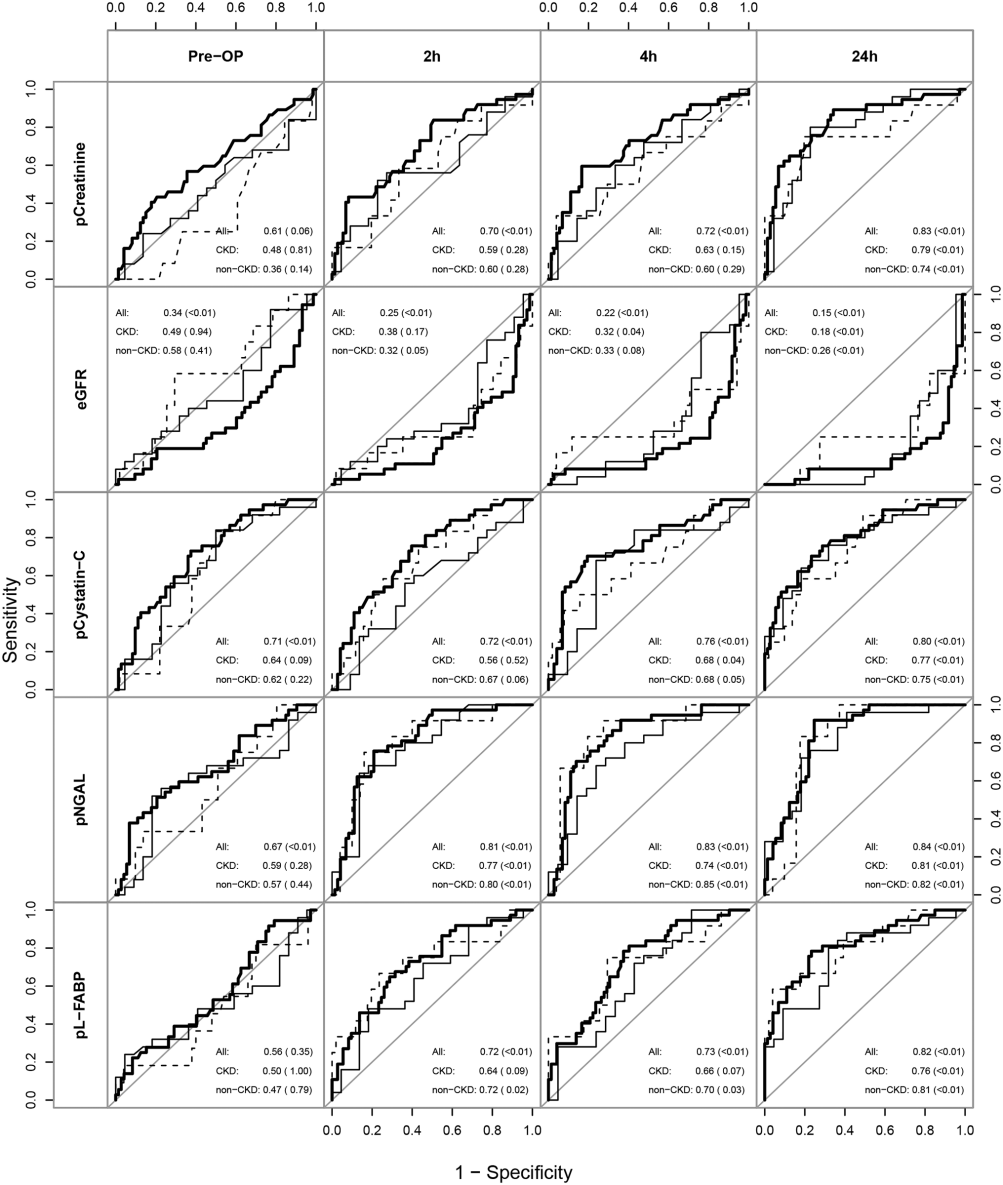
Diagnostic performance of biomarkers in plasma. Receiver operating characteristic curves (AUC-ROC) show discriminative abilities for preoperative risk stratification (pre-OP) and postoperative diagnosis of AKI (at 2h, 4h and 24h after CPB) for all patients (solid thick line) and separately for patients with (solid thin line) and without (dashed line) preexisting CKD. Indicated are AUC (p-value).

First, we analyzed the time course of standard renal function parameters and novel biomarkers for AKI in plasma samples. With the exception of urinary creatinine and NGAL, normal ranges of AKI biomarkers in plasma and urine were significantly, 21% to 196% higher in non-AKI patients before surgery in comparison to healthy volunteers (S1 Fig). However, cohorts of healthy individuals and cardiac surgery patients were not matched for sex and age. In AKI patients, plasma creatinine and cystatin C levels increased until the third postoperative day, whereas they remained stable in non-AKI patients. In contrast plasma NGAL and L-FABP already peaked within 4h after CPB in AKI patients (Fig 1). ROC curve analysis showed that plasma NGAL had the best diagnostic performance early (within 24 hours) after surgery: mean AUC values were 0.81 (95% confidence interval (CI) 0.73–0.90) at 2h and 0.83 (0.75–0.91) at 4h after CPB (Fig 2). AUC values of biomarkers in plasma increased with extending duration after CPB. After 24h plasma NGAL and L-FABP did not perform better than plasma creatinine and cystatin C to detect AKI. Cutoff values of plasma and urinary biomarkers were calculated for a specificity fixed by 75% (S2 Table).

Moreover, 6 intraoperative variables (type and duration of surgery, CPB, aorta disconnection, reperfusion, and mechanical ventilation time) were evaluated for their association with postoperative plasma biomarker levels by calculating rank correlation coefficients. We found merely weak correlations of plasma NGAL levels 2h and 4h after CPB with the duration of surgery (0.26, 0.20), CPB (0.23, 0.16), aortic cross-clamping (0.24, 0.22) and mechanical ventilation (0.41, 0.38).

### Early diagnosis of postoperative AKI with biomarkers in urine

**Fig 3:**
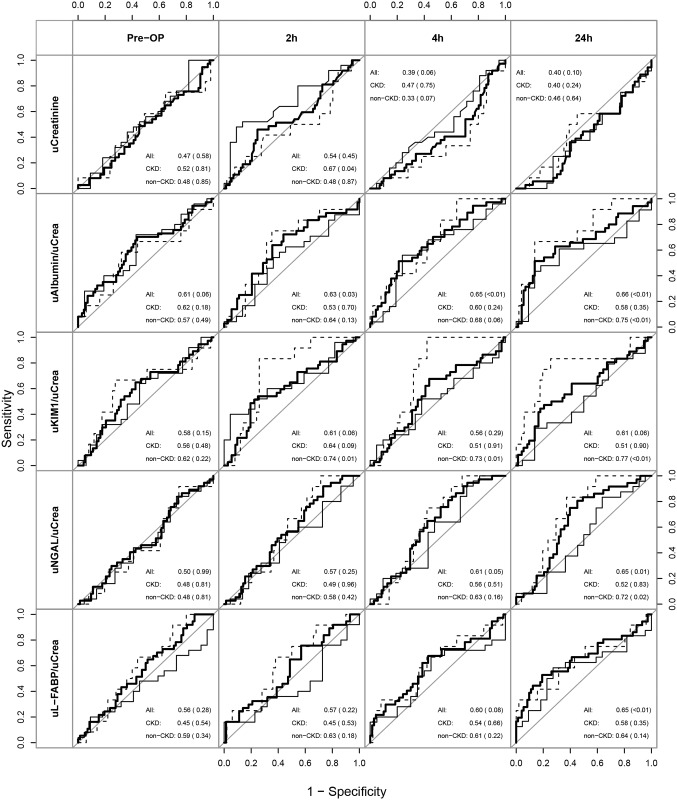
Diagnostic performance of biomarkers in urine. Receiver operating characteristic curves (AUC-ROC) show discriminative abilities for preoperative risk stratification (pre-OP) and postoperative diagnosis of AKI (at 2h, 4h and 24h after CPB) for all patients (solid thick line) and separately for patients with (sold thin line) and without (dashed line) preexisting CKD. Indicated are AUC (p-value).

Next, we analyzed urinary levels of the same biomarkers measured in plasma (when applicable) in direct comparison at corresponding time points. Urinary creatinine levels significantly declined 2h after surgery compared to preoperative levels in AKI and non-AKI patients, most likely due to intraoperative volume resuscitation (Fig 1). None of the urine biomarkers albumin, NGAL, L-FABP, and KIM1 was discriminative for AKI when considered as absolute concentration per ml. Normalization of urine biomarkers to urinary creatinine resulted in slightly better, albeit still poor performance. Postoperative urinary NGAL, L-FABP, and KIM1 did not differentiate AKI and non-AKI patients (Fig 1), whereas postoperative urinary albumin/creatinine ratio (ACR) detected AKI with an AUC [95% CI] of 0.63 [0.51–0.74] at 2h and 0.65 [0.55–0.76] at 4h after CPB (Fig 3).

### Diagnostic performance of additional biomarkers in plasma and urine

**Fig 4:**
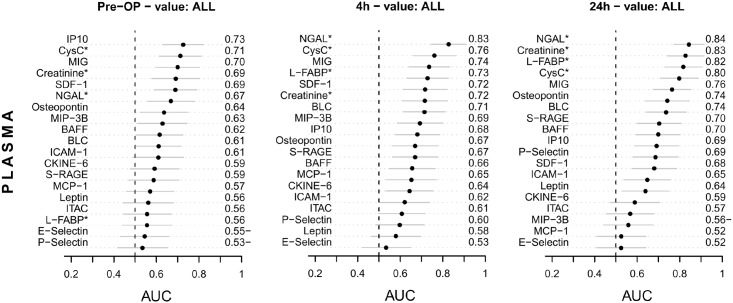
Ranking of candidate plasma biomarkers. Ranking of biomarkers in plasma according to their preoperative (pre-OP) and postoperative (4h and 24h after CPB) AUC-ROC performance for risk stratification and detection of AKI. AUC values <0.5 were expressed as 1-AUC indicated by AUC−. Parameters marked with * were measured with the assays indicated in the methods section, all others were measured using the Human CustomMAP. The confidence intervals for AUCs were calculated with the DeLong method. Abbreviations: BAFF, B-cell activating factor; BLC, B lymphocyte chemoattractant, chemokine C-X-C motif ligand (CXCL) 13; CKINE-6, chemokine with 6 cysteines, chemokine C-C motif ligand (CCL) 21; CysC, cystatin C; ICAM-1, intercellular adhesion molecule 1, cluster of differentiation (CD) 54; IP10, interferon-γ-induced protein 10, CXCL 10; ITAC, interferon-inducible T-cell alpha chemoattractant, CXCL11, IP9; L-FABP, liver-type fatty acid-binding protein; MCP-1, monocyte chemotactic protein-1, CCL2; MIG, monokine induced by interferon-γ, CXCL9; MIP3B, macrophage inflammatory protein-3ß, CCL19; NGAL, neutrophil gelatinase-associated lipocalin; SDF-1, stromal cell-derived factor-1, CXCL12; S-RAGE, soluble receptor for advanced glycosylation end products.

**Fig 5:**
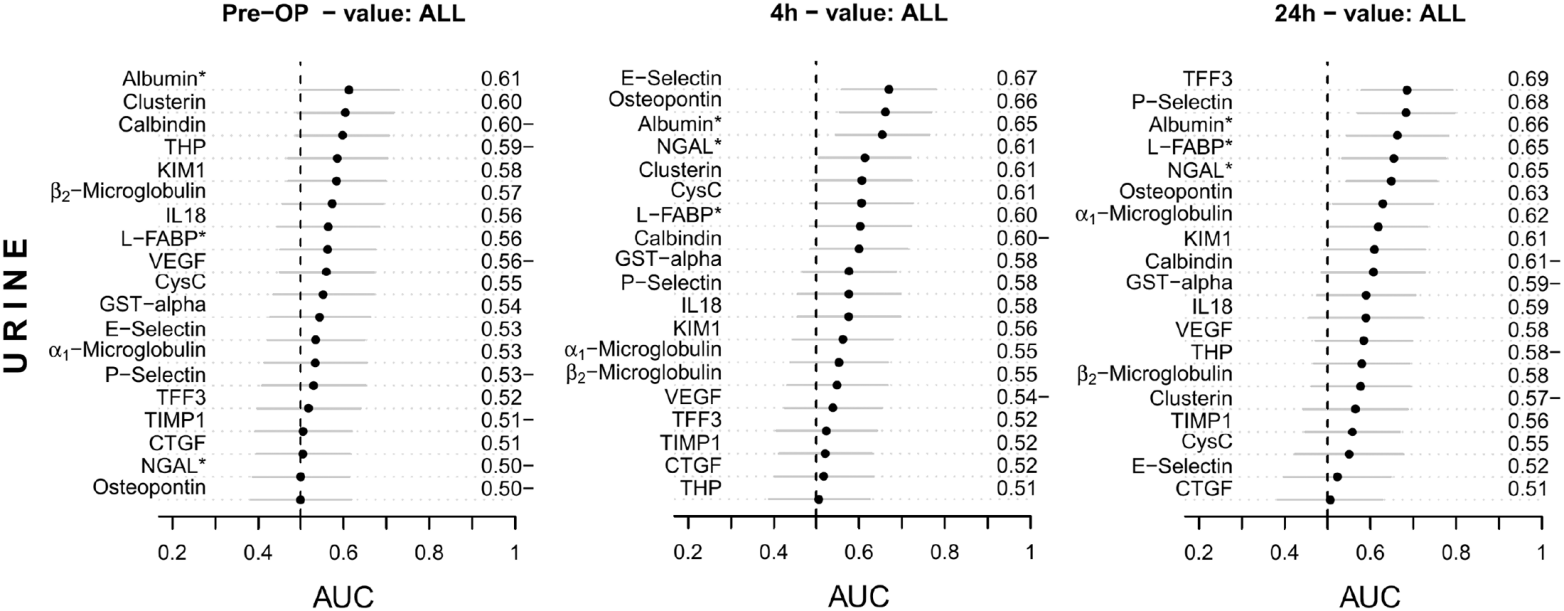
Ranking of candidate urine biomarkers. Ranking of biomarkers in urine according to their preoperative (pre-OP) and postoperative (4h and 24h after CPB) AUC-ROC performance for risk stratification and detection of AKI. AUC values <0.5 were expressed as 1-AUC indicated by AUC−. Parameters marked with * were not included in the Human KidneyMAP^®^ and were measured separately. The confidence intervals for AUCs were calculated with the DeLong method. Abbreviations: CTGF, connective tissue growth factor; CysC, Cystatin C; GSTα, glutathione S-transferase-α; IL18, interleukin 18; KIM1, kidney injury molecule 1; L-FABP, liver-type fatty acid-binding protein; NGAL neutrophil gelatinase-associated lipocalin; THP, Tamm-Horsfall protein; TIMP1, tissue inhibitor of metalloproteinases 1; TFF3, trefoil factor 3; VEGF, vascular endothelial growth factor.

A recent meta-analysis identified NGAL as the biomarker with best discriminative performance for early diagnosis of cardiac surgery associated AKI and for outcome prediction [4]. Therefore we assessed an array of biomarkers in plasma and urine using the multiplex immunoassays Human CustomMAP (Fig 4) and Human KidneyMAP^®^ (Fig 5) respectively before, 4h and 24h after surgery and compared its diagnostic performance to NGAL.

Postoperatively none of the additional 15 plasma biomarkers (Fig 4) outperfomed plasma NGAL, which had the best AUC values for AKI detection 4h and 24h after surgery (AUC 0.83 and 0.84). At 4h plasma NGAL was followed by cystatin C, monokine induced by interferon-γ (MIG) and L-FABP (AUC 0.76–0.73), which also had greater AUC values than plasma creatinine (0.72), however the difference was not statistically significant. After 24h none of the 15 plasma biomarkers of the CustomMAP panel performed better than plasma creatinine or NGAL.

In corresponding urine samples biomarkers had overall lower AUCs to detect AKI than plasma biomarkers, and no KidneyMAP^®^ marker in urine had higher AUC values than plasma creatinine or NGAL at any time point (Fig 5). However, 4h after surgery E-selectin, osteopontin, and albumin, at 24h trefoil factor 3 (TFF3), P-selectin, albumin, L-FABP, and NGAL were at top of urinary biomarkers for AKI detection (all AUC vales ≤0.69). Also urinary IL-18, which had good discriminative performance mainly in pediatric cardiac surgery populations [28–30], was overall not discriminative in our cohort (AUC 0.69 at 2h, 0.58 at 4h, 0.59 at 24h).

We also analyzed correlation of each plasma and urine biomarker with plasma creatinine using Spearman's rank correlation coefficients. Plasma creatinine correlated best with plasma NGAL and cystatin C as well as urinary calbindin and uromodulin (Tamm-Horsfall protein) before as well as 4h and 24h after cardiac surgery (S3 Table).

### Plasma and urine biomarkers to identify patients at risk for AKI

Next, we evaluated if preoperative biomarkers in plasma or urine could predict patients at risk for postoperative AKI. Highest AUC values for AKI risk stratification had plasma interferon γ-induced protein 10 (IP10) (0.73), cystatin C (0.71), and monokine induced by interferon-γ (MIG) (0.70) (Fig 4), although these results were not statistically different from plasma creatinine (0.69). Most urine biomarkers showed lower AUC values than biomarkers in plasma to predict the risk for AKI. The best biomarker in urine for AKI risk prediction was preoperative urinary ACR with an AUC of 0.61 (Fig 5).

### Combination of biomarkers with clinical scores for AKI risk stratification

In addition, we tested the discriminative ability of preoperative biomarkers for AKI risk stratification, when they were added to a clinical model. Among available preoperative clinical scoring systems the CCF score offers the best discriminative power to predict dialysis-requiring AKI [31–34]. EuroSCORE was originally developed to predict the perioperative mortality risk following cardiac surgery, but it also showed very good discriminatory ability in predicting postoperative AKI [35,36]. In our cohort EuroSCORE defined 73% of AKI patients as high risk, and none of the patients belonging to the low risk group developed AKI (Table 2). By contrast, the CCF score classified only 16% of AKI patients as having high risk (Table 2). AUC values were 0.76 and 0.71 for EuroSCORE and CCF score respectively to predict the risk for AKI (Table 3). The lower discriminative power of CCF score in comparison to EuroSCORE might at least in part be explained by less included attributes.

**Table 2:** AKI risk stratification according to EuroSCORE and CCF Score.

		EuroSCORE		CCF Score	
Score Points	Risk	Non-AKI	AKI	Non-AKI	AKI
0–2	Low	14 (19%)	0 (0%)	34 (47%)	8 (22%)
3–5	Medium	36 (49%)	10 (27%)	36 (49%)	23 (62%)
>5	High	23 (32%)	27 (73%)	3 (4%)	6 (16%)

**Table 3:** Combinations of preoperative biomarkers and clinical scores to predict the risk for AKI.

	EuroSCORE			CCF score		
	AUC	p	Sensitivity	AUC	p	Sensitivity
**Score alone**	0.76	<0.001	0.58 (0.34,0.81)	0.71	<0.001	0.57 (0.37,0.73)
**Score + pCreatinine**	0.78	<0.001	0.68 (0.35, 0.86)	0.71	<0.001	0.57 (0.32, 0.78)
**Score + pNGAL**	0.80	<0.001	0.65 (0.41,0.86)	0.76	<0.001	0.68 (0.43,0.84)
**Score + pIP10**	0.80	<0.001	0.64 (0.42,0.89)	0.75	<0.001	0.61 (0.44,0.78)
**Score + pCystatin C**	0.80	<0.001	0.65 (0.46,0.84)	0.74	<0.001	0.65 (0.49,0.81)
**Score + uACR**	0.78	<0.001	0.59 (0.38,0.81)	0.74	<0.001	0.62 (0.41,0.78)
**Score + uKIM1**	0.77	<0.001	0.65 (0.35,0.84)	0.72	<0.001	0.57 (0.38,0.81)
**Score + pL-FABP**	0.76	<0.001	0.64 (0.36,0.83)	0.74	<0.001	0.67 (0.44,0.83)
**Score + uNGAL**	0.76	<0.001	0.57 (0.32,0.81)	0.72	<0.001	0.57 (0.35,0.73)
**Score + uL-FABP**	0.75	<0.001	0.62 (0.32,0.81)	0.70	<0.001	0.54 (0.35,0.73)

Biomarkers in urine are normalized to urinary creatinine. The column “sensitivity” (given as mean and 95% confidence intervals) contains sensitivity of combination for specificity fixed by 75%. The 95% confidence intervals for sensitivity were calculated using bootstrap method using R-Package pROC (version 1.5). Abbreviations: ACR, albumin/creatinine ratio; AUC, area under the curve; CCF, Cleveland Clinic Foundation; EuroSCORE, European System for Cardiac Operative Risk Evaluation; IP10, interferon-γ-induced protein 10; KIM1, kidney injury molecule 1; L-FABP, liver fatty acid binding protein; NGAL, neutrophil gelatinase-associated lipocalin; p, plasma; u, urine.

When preoperative biomarkers were combined with one of the clinical models, their predictive performance increased. The improvement was better for the combination with EuroSCORE than with CCF score. In our cohort the best combination to predict risk for AKI was EuroSCORE plus preoperative plasma NGAL or cystatin C or IP10 (all with an AUC of 0.80) (Table 3). However, the difference in AUC between the combination and EuroSCORE or biomarker alone was not significant. Urine biomarkers could not improve the predictive performance of clinical scores, when they were used in combination.

### Multivariable analysis of plasma and urine biomarkers

Combining multiple biomarkers has been shown to increase their accuracy and reliability for the diagnosis and/or risk stratification of AKI patients [5,29,37–39]. Therefore we built a multivariable model by using generalized linear regression with lasso regularization and evaluated its performance with 10-fold cross validation. We included plasma biomarker values at baseline and 2h after surgery. Lasso logistic regression did not yield a combination of 2 or more biomarkers in plasma and urine with consistently better performance than plasma NGAL alone. Considering only urine markers resulted in an even larger model whose AUC still was only around 0.68.

### Impact of preexisting CKD on plasma and urine biomarkers

In our cohort 53% of AKI patients had preexisting CKD (stage ≥2), while 81% of patients without AKI did not have CKD (Table 1). Before surgery CKD patients had significantly higher plasma levels of creatinine, cystatin C, NGAL, L-FABP as well as urinary levels of L-FABP (S2 Fig).

If AKI biomarkers showed fundamental differences in CKD and non‐CKD patients, they might separate overall groups (CKD and non‐CKD) while not really being diagnostic for AKI. Therefore we also performed a separate ROC analysis stratified for baseline kidney function (Figs 2 and 3). After surgery AUC values for urinary ACR and KIM1 as well as plasma and urinary NGAL and L-FABP to detect AKI were lower in CKD compared to non-CKD patients. However, plasma NGAL remained the best biomarker for AKI detection in non-CKD as well as CKD patients. In contrast, before surgery plasma NGAL, L-FABP, cystatin C, and urinary ACR had higher AUC values for AKI risk prediction in CKD than in non-CKD patients.

Among the additional biomarkers we measured in plasma and urine using multiplex assays, AUC values for plasma (S3 Fig) and urine biomarkers (S4 Fig) were overall lower in CKD than in non-CKD patients, even though the pattern was heterogeneous.

## Discussion

We studied a cohort of 110 unselected, adult patients undergoing cardiac surgery with CPB. Within 3 days after surgery 37 patients developed AKI. The number of patients and AKI events in our cohort match with median values in a recent meta-analysis on AKI biomarkers after adult cardiac surgery [17]. In comparison to the multicenter cohort study TRIBE-AKI [5], our single-center cohort included more male patients with higher prevalence of hypertension, congestive heart failure, presurgical cardiac catherization, ACE inhibitor or ARB medication, and worse preoperative kidney function. AKI patients in our study were mainly assigned to AKIN stage 1, corresponding to the “mild” AKI subgroup in the TRIBE-AKI cohort. Therefore results of our study will mostly reflect mild AKI, although we did not stratify our results for mild (AKIN stage 1) and severe forms (AKIN stage ≥2) of AKI.

### Plasma and urine biomarker biology

Renal biomarkers are being evaluated in plasma and/or urine samples with still numerous unanswered questions and uncertainties. Inconsistent and sometimes contradictory findings in clinical studies might also be attributed to different biology of biomarkers in plasma and urine, which can be representatively described for NGAL. Following different types of insults to the kidneys intrarenal NGAL expression is upregulated especially in the distal nephron indicating that NGAL may be secreted into the urine [40–42]. At least *in vitro* also proximal tubular cells have been shown to secrete NGAL in response to ATP depletion [43]. After AKI plasma NGAL also increases, and NGAL is freely filtered in the glomeruli and reabsorbed in the proximal tubule by megalin-dependent endocytosis [41,44]. Thus increased plasma NGAL in AKI may result from tubular backleak, extrarenal origin (potentially totally independent from kidney disease), and/or reduced glomerular filtration. Elevated urinary NGAL may reflect induced renal expression, glomerular filtration of plasma NGAL from renal or extrarenal sources and/or impaired tubular reabsorption [45,46]. Leukocyturia in the setting of urinary tract infections also leads to increased urinary NGAL levels [47]. Vanmassenhove et al. speculated that urinary markers might be more sensitive for true histological damage, whereas serum levels of markers might be more sensitive for changes in clearance [4]. Since we found a better correlation of plasma creatinine with plasma biomarkers than with urinary biomarkers, our data might support this concept (S3 Table). In line with this assumption, in septic patients plasma markers might rather reflect overall disease severity and multiorgan failure than kidney damage only [4,48]. However, the source and fate of biomarkers are not yet fully established and need further investigation.

In contrast to urine, plasma is easily obtainable for biomarker assessment even in oligo-/anuric patients. On the one hand plasma biomarkers seem to be dispensable in oliguric patients, as oliguria is a diagnostic and staging criterion for AKI, and it was also shown to be an early and sensitive, albeit non-specific predictor of biochemical AKI [49,50]. On the other hand plasma biomarkers might help to guide fluid therapy in oliguric patients while maneuvering between continued fluid resuscitation to reverse incipient organ dysfunction and avoidance of harmful fluid overload [51]. Plasma biomarkers might also help to assess severity and prognosis of AKI in oliguric patients. So plasma biomarkers might indeed have a therapeutic and prognostic role in oliguric AKI patients and might obviously have practical advantages over urine biomarkers.

#### AKI diagnosis

We measured plasma and/or urine levels of cystatin C, NGAL, L-FABP, KIM1, and albumin and assessed their capability for early (within 24h after surgery) diagnosis of AKI. In line with previous findings plasma and/or urine biomarkers were significantly elevated in AKI in comparison to non-AKI patients and differentiated AKI and non-AKI patients earlier than plasma creatinine or cystatin C. The discriminative performance (based on AUC-ROC analyses) of biomarkers for detection of AKI increased with time after CPB, as previously described [52,53], but their advantage over plasma creatinine was better early after CPB (2 and 4h).

Biomarkers had shown better diagnostic performance in severe than in mild forms of AKI [5,54], which predominated in our cohort. Overall recent studies in heterogeneous adult patients reported less effective diagnostic performance of biomarkers [4,17] than in the initial studies in children without confounding co-morbidities (e.g. CKD) after CPB [55–57].

Our results are largely consistent with findings in the TRIBE-AKI study despite our smaller patient cohort, differences in the time points of sample collection and the standardization of urinary biomarker levels (S4 Table). In our study plasma NGAL, cystatin C, and L-FABP showed the best discriminative values to detect AKI. We found better AUC values for plasma NGAL (AUC >0.8) than TRIBE-AKI investigators [5] and a recent meta-analysis [17]. In our study plasma cystatin C had similar diagnostic performance compared to recent studies meta-analyzed in [17]. Up to now only 2 studies described increased plasma L-FABP levels in patients with AKI after pediatric cardiac surgery [58] and adult renal transplantation [59]. The relationship between plasma and urinary L-FABP might be similar to NGAL as outlined above. However, the mechanism underlying the increase in plasma L-FABP levels remains unclear.

In contrast to previous investigations [5,29,37–39], in our cohort combination of biomarkers did not improve their diagnostic performance, when plasma NGAL was included in any combination. However, in the TRIBE-AKI study combinations of biomarkers with plasma NGAL also improved the diagnostic performance only slightly [5,8].

In comparison to postoperative clinical models (incorporating pre-, peri- and early postoperative risk factors) for identification of AKI patients after cardiac surgery, in our study pNGAL performed better than the clinical model used by Parikh et al. [5] (AUC 0.69), but showed similar discriminative performance to the clinical models developed by Palomba et al. [60] (0.85) and Ng et al. [61] (0.81). However, only the TRIBE-AKI study [5] defined postoperative AKI by the consensus AKIN criteria.

When we tested additional plasma biomarkers, only plasma levels of the chemokine monokine induced by IFN-γ (MIG, also known as CXC ligand 9, CXCL9) reached a moderate AUC value for detecting AKI. Plasma [62] and urinary MIG [63,64] have been investigated as indicators of acute renal allograft rejection.

We found lower AUC values for urinary KIM1 in comparison to the TRIBE-AKI cohort, whereas the diagnostic performance of urinary ACR, cystatin C, IL-18, L-FABP, and NGAL was similar (S4 Table) [5,8,65,66]. In direct comparison our study demonstrated less discriminative performance to detect postoperative AKI for urinary than for plasma NGAL (S5 Fig).

Interestingly, in our study biomarkers measured in urine were overall less effective to detect AKI than plasma biomarkers. Standardization of urine biomarkers to urinary creatinine was necessary to increase their diagnostic performance. The validity of normalizing urine biomarkers for urinary creatinine is still controversial in the literature [5,6,9,13–16]. Our results indicate that in patients with the need of high fluid resuscitation (e.g. major surgery, sepsis) standardization of urine biomarkers might be required to account for variations of urinary flow rate. Moreover, urine specimen types and collection procedures differed in various clinical studies potentially influencing biomarker results. However, the reason for limited diagnostic performance of urine biomarkers in our study remains elusive. Although differences in AUC values between plasma and urine biomarkers did not reach statistical difference, most likely due to the limited size of our cohort, our results are still of clinical interest and suggest to rely on plasma rather than urine markers for diagnostic purposes.

#### AKI risk stratification

Renal biomarkers measured in plasma and/or urine before surgery may help to identify patients at risk for postoperative AKI. In our study preoperative plasma IP10, cystatin C, and MIG had higher AUC values for risk stratification of AKI than plasma creatinine, which was insignificantly increased in AKI compared to non-AKI patients before surgery. Preoperative cystatin C was significantly higher in AKI than in non-AKI patients and had an AUC value of 0.71 to predict risk for AKI, which is in line with recent publications [67,68]. Interestingly, the proinflammatory chemokines IP10 (also known as CXCL10) and MIG (CXCL9) also had moderate AUC values to predict the risk for AKI. These findings might imply that a preexisting inflammatory state before surgery may promote development of postoperative AKI, as the inflammatory response is one of the important contributors for renal dysfunction after cardiac surgery [69]. In line with this hypothesis preoperative C-reactive protein (CRP) [70] and macrophage inflammatory protein (MIP)-1β [71] also predicted risk for AKI after cardiac surgery in adults.

Among urine biomarkers preoperative urinary ACR correlated with the later development of AKI, as it has been shown in larger cohorts as well [72,73].

Combining preoperative biomarkers with clinical models has been described to increase their performance to identify patients at risk for AKI after cardiac surgery [74]. In our study addition of neither urine nor plasma biomarkers to clinical scores (EuroSCORE, CCF score) significantly improved their discriminative performance. This might be mainly due to the fact that the AUC value of the EuroSCORE model (0.76) was already higher than the Society of Thoracic Surgeons’ risk model used in the TRIBE-AKI study (0.70 for “mild”, 0.73 for “severe” AKI) [74]. Of note, both models are presurgical risk assessment tools for perioperative mortality [75], not validated for acute kidney injury with or without requirement of RRT. However, they include many patient-related risk factors for AKI associated with cardiac surgery [69], and thus they may reflect the patients’ susceptibility to AKI after cardiac surgery. Our results highlight the utility and importance of preoperative risk stratification using clinical scoring systems in patients undergoing cardiac surgery, which were not inferior to biochemical AKI markers in our study. Therefore clinical models might be preferred to biochemical markers for preoperative risk stratification of AKI. However, a validated score to predict (non-)dialysis requiring AKI is lacking, so that further studies are required to validate established and/or develop new risk models for prediction of mild AKI after cardiac surgery.

### CKD impact on AKI biomarkers

In our study preexisting CKD predisposed for AKI after cardiac surgery, which is in line with previous studies reporting CKD as an independent risk factor for AKI (and vice versa) [76]. Understanding of AKI biomarkers in CKD patients is still very incomplete, which can again be representatively demonstrated for NGAL. Although NGAL is highly induced by triggers of AKI, elevated (plasma and urinary) NGAL levels have also been reported in adults with progressive CKD [77,78] blurring the differential diagnosis between CKD and AKI. However, NGAL is only marginally increased during periods of slowly progressive CKD [79–81]. Increase of NGAL in CKD might be the consequence of reduced renal clearance and/or sustained production by “burning” tubular cells reflecting active kidney damage (“forest fire hypothesis”) [82]. Indeed, in our study we found significantly elevated baseline levels not only of plasma NGAL but also of plasma cystatin C, L-FABP and urinary L-FABP in CKD compared to non-CKD patients. However, we cannot exclude that CKD patients had superimposed AKI before surgery e.g. due to preoperative cardiac catheterization.

The role of AKI biomarkers in CKD is complicated by the fact that in CKD patients the percentage increase in plasma creatinine used to define AKI generally occurs later compared to patients with previously normal renal function, and thus, defining AKI using only plasma creatinine criteria could diminish the sensitivity of AKI diagnosis in CKD patients [83]. Consequently, current AKI classification systems show better performance in patients without CKD [84].

In our study AKI biomarkers had overall poorer diagnostic performance in CKD patients in comparison to patients without CKD. This is in line with previous observations [53,85–88], and might result from impaired and/or more variable biomarker excretion in CKD.

Indeed, the poor performance of AKI biomarkers in patients with pre-existing CKD still limits their utility at the moment. This is particularly problematic in view of the high prevalence and incidence of CKD [89] and the high risk for AKI in CKD patients [76]. Hitherto, only few studies have addressed this problem. Further studies are needed to establish differential cutoff values for AKI biomarkers among patients with and without CKD, to understand the behavior of AKI biomarkers in CKD patients, to validate present AKI biomarkers for selected comorbidities (in particular CKD), and to identify new biomarkers for differentiation of AKI and CKD. Until then serial biomarker measurements might be a compromise in patients with CKD or other comorbidities.

### Limitations

We recognize that our study is limited by its single-center recruitment. Thus results might be influenced by therapeutic algorithms specific to our center, which may not be applicable to other centers. Our cohort included a higher proportion of patients with mild AKI stages compared to previous studies, which might influence biomarker performance. Although we identified several (plasma) biomarkers with better AUC values than plasma creatinine for risk stratification and early diagnosis of AKI, results did not reach statistical significance, which may be, at least in part, due to the small sample size of our cohort. Therefore further studies are needed to confirm our findings.

### Conclusions

We compared the discriminative performance of renal biomarkers in plasma and urine for risk stratification and early diagnosis of AKI in unselected adult patients undergoing cardiac surgery with CPB. AUC-ROC analyses indicated overall better diagnostic performance of biomarkers in plasma than in urine. Normalization of urine biomarkers to urinary creatinine was essential for AKI risk stratification and diagnosis after cardiac surgery, but AUC values of normalized urine biomarkers still lagged behind those of plasma biomarkers. Plasma NGAL had the best AUC value for early diagnosis of AKI after surgery. Biomarker combinations did not improve the discriminative power of plasma or urine biomarkers. For preoperative AKI risk stratification clinical scoring systems (CCF score and particularly EuroSCORE) were similarly effective as biomarkers. The discriminative performance of both plasma and urine biomarkers was limited by preexisting CKD.

## Supporting Information

S1 Fig Normal reference ranges for AKI biomarkers in plasma and urine of healthy volunteers. Scatter plots of biomarker values with median and interquartile range of healthy volunteers (controls, n = 30) and non-AKI study patients before surgery (n = 73). Biomarkers in urine are normalized to urinary creatinine (Crea). *, p < 0.05.
(PDF)

S2 Fig Preoperative plasma and urinary levels of AKI biomarkers stratified for CKD and non-CKD patients. Scatter plots of preoperative biomarker values with median and interquartile range for CKD (n = 47) and non-CKD (n = 63) patients. Biomarkers in urine are normalized to urinary creatinine (Crea). *, p < 0.05.
(PDF)

S3 Fig Ranking of candidate plasma biomarkers in CKD and non-CKD subgroups. Ranking of biomarkers in plasma according to their preoperative (pre-OP) and postoperative (4h and 24h after CPB) AUC-ROC performance for risk stratification and early diagnosis of AKI stratified in CKD and non-CKD subgroups. AUC values <0.5 were expressed as 1-AUC indicated by AUC−. Parameters marked with * were measured with the assays indicated in the methods section, all others were measured using the Human CustomMAP. The confidence intervals for AUCs were calculated with the DeLong method. Abbreviations: BAFF, B-cell activating factor; BLC, B lymphocyte chemoattractant, chemokine C-X-C motif ligand (CXCL) 13; CKINE-6, chemokine with 6 cysteines, chemokine C-C motif ligand (CCL) 21; CysC, cystatin C; ICAM-1, intercellular adhesion molecule 1, cluster of differentiation (CD) 54; IP10, interferon-γ-induced protein 10, CXCL 10; ITAC, interferon-inducible T-cell alpha chemoattractant, CXCL11, IP9; L-FABP, liver-type fatty acid-binding protein; MCP-1, monocyte chemotactic protein-1, CCL2; MIG, monokine induced by interferon-γ, CXCL9; MIP3B, macrophage inflammatory protein-3ß, CCL 19; NGAL, neutrophil gelatinase-associated lipocalin; SDF-1, stromal cell-derived factor-1, CXCL12; S-RAGE, soluble receptor for advanced glycosylation end products.
(PDF)

S4 Fig Ranking of candidate urine biomarkers in CKD and non-CKD subgroups. Ranking of biomarkers in urine according to their preoperative (pre-OP) and postoperative (4h and 24h after CPB) AUC-ROC performance for risk stratification and early diagnosis of AKI stratified in CKD and non-CKD subgroups. AUC values <0.5 were expressed as 1-AUC indicated by AUC−. Parameters marked with * were not included in the Human KidneyMAP^®^ and were measured separately. The confidence intervals for AUCs were calculated with the DeLong method. Abbreviations: CTGF, connective tissue growth factor; CysC, Cystatin C; GSTα, glutathione S-transferase-α; IL18, interleukin 18; KIM1, kidney injury molecule 1; L-FABP, liver-type fatty acid-binding protein; NGAL neutrophil gelatinase-associated lipocalin; THP, Tamm-Horsfall protein; TIMP1, tissue inhibitor of metalloproteinases 1; TFF3, trefoil factor 3; VEGF, vascular endothelial growth factor.
(PDF)

S5 Fig ROC curves for plasma and urinary NGAL to detect postoperative AKI 4 hours after CPB. AUC values are given as mean (95% confidence intervals). Urinary NGAL is normalized to urinary creatinine.
(PDF)

S1 Table Additional characteristics of study participants stratified by AKI. For categorical variables the first column (“All”) shows column percentages. The columns “non-AKI” and “AKI” contain row percentages. Abbreviations: ACE, angiotensin converting enzyme; ARB, angiotensin receptor blocker; ICU, intensive care unit; IMC, intermediate care unit; max, maximum; min, minimum.
(PDF)

S2 Table Test characteristics of plasma and urinary AKI biomarkers. Sensitivities (given as point estimates and 95% confidence intervals) and cutoff values of plasma and urinary biomarkers were determined from the AUC curves in Figs 2 and 3 setting the specificity to 75%. Biomarkers in urine are normalized to urinary creatinine (Crea).
(PDF)

S3 Table Correlation of plasma creatinine with plasma and urinary AKI biomarkers. The relationship between plasma creatinine and various (A) plasma and (B) urinary AKI biomarkers at indicated time points was analyzed by Spearman’s rank correlation coefficient. All biomarkers in urine are normalized to urinary creatinine.
(PDF)

S4 Table AUC-ROC analyses for early diagnosis of AKI with renal biomarkers after adult cardiac surgery in comparison to recent studies from the literature. In this study AKI was defined by the AKIN serum creatinine criteria within 72h after surgery. In the TRIBE-AKI study “severe” AKI was defined as AKIN stage ≥2 based on serum creatinine criteria during the entire hospital stay [5,8,65,66]. “Mild” AKI was defined either as RIFLE stage “R” serum creatinine criteria or requirement of acute dialysis [5] or AKIN stage 1 serum creatinine criteria during the entire hospital stay [65,66]. In this study plasma and urine samples were collected 2 or 4h after discontinuation of CPB, whereas in the TRIBE-AKI study first postoperative samples (0- to 6-hour time point) were taken at a median of 0.25 (IQR 0.1 to 0.5) hours after arrival to the ICU. In contrast to the TRIBE-AKI study, in this study urine biomarkers were normalized to urinary creatinine. Data from the TRIBE-AKI study were summarized from [5,8,65,66].
(PDF)
