# Supplementary figures and images for: Comparison of Plasma and Urine Biomarker Performance in Acute Kidney Injury
Source: PLoS One. 2015 Dec 15;10(12):e0145042. doi: 10.1371/journal.pone.0145042 (PMC4682932; doi:10.1371/journal.pone.0145042)

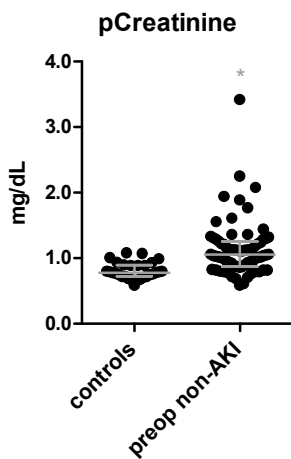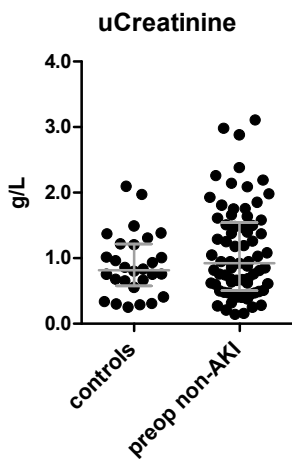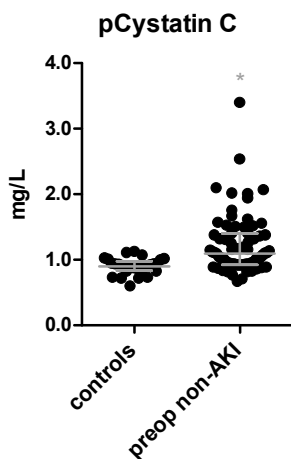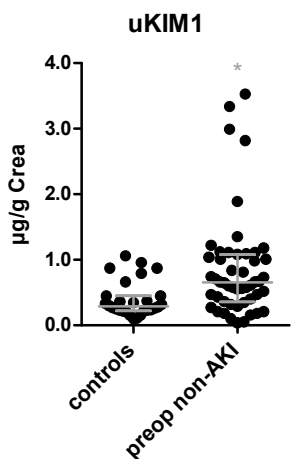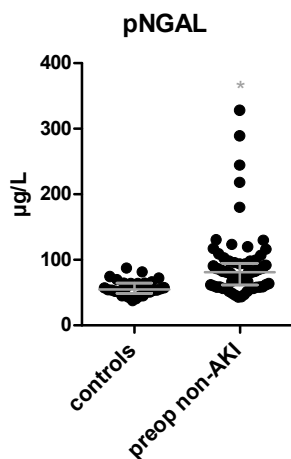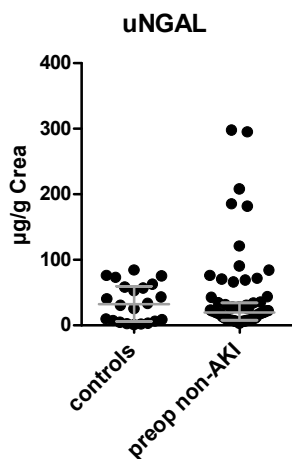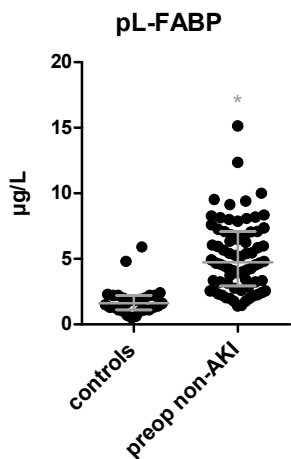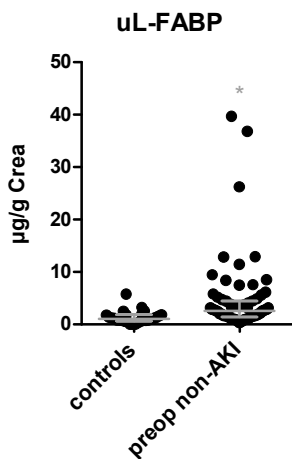

Supplement: S1 Fig — Scatter plots of biomarker values with median and interquartile range of healthy volunteers (controls, n = 30) and non-AKI study patients before surgery (n = 73). Biomarkers in urine are normalized to urinary creatinine (Crea). *, p < 0.05. (PDF) [file pone.0145042.s001.pdf]

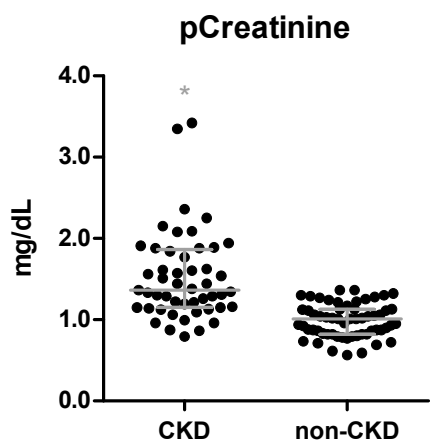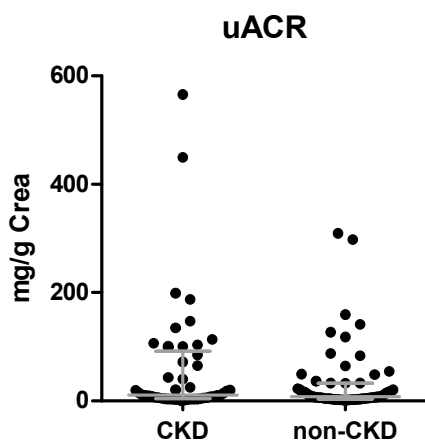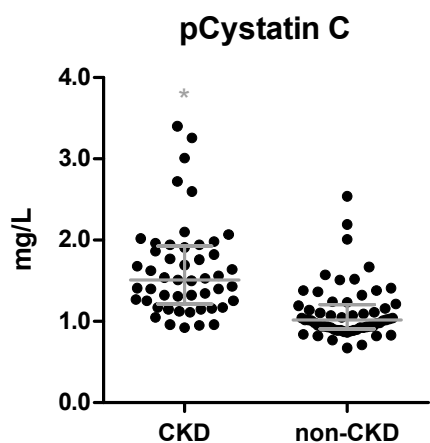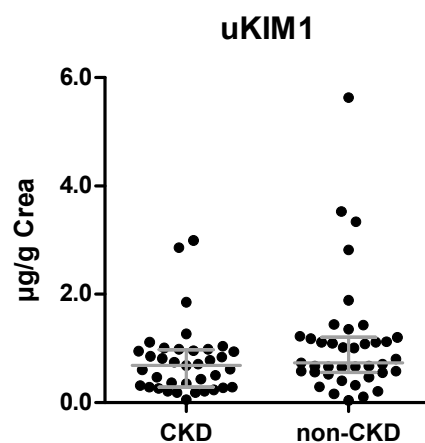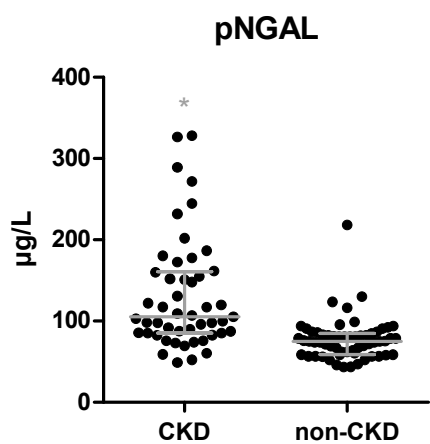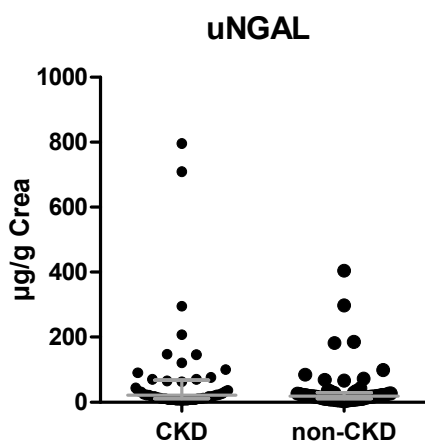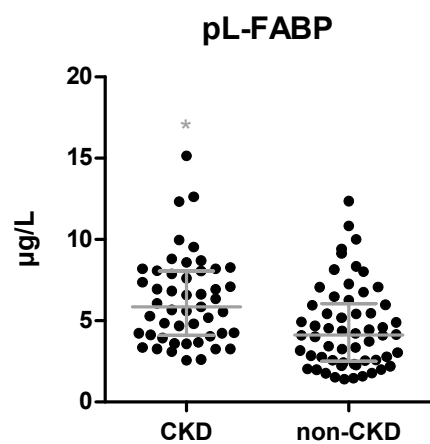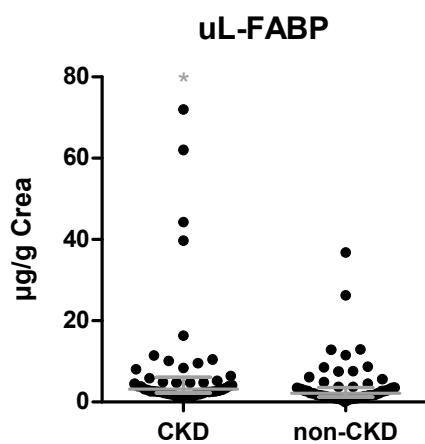

Supplement: S2 Fig — Scatter plots of preoperative biomarker values with median and interquartile range for CKD (n = 47) and non-CKD (n = 63) patients. Biomarkers in urine are normalized to urinary creatinine (Crea). *, p < 0.05. (PDF) [file pone.0145042.s002.pdf]

Pre-OP – value: CKD

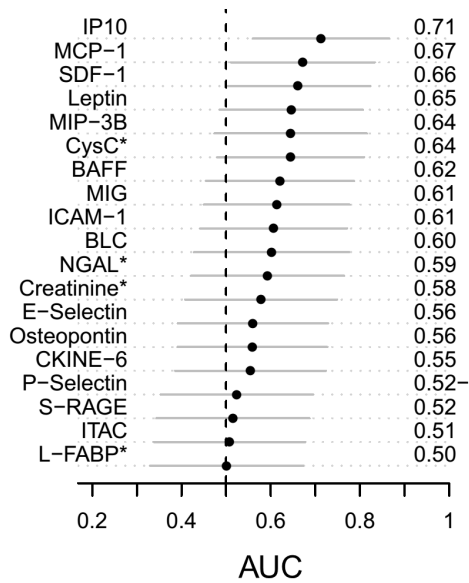

4h – value: CKD

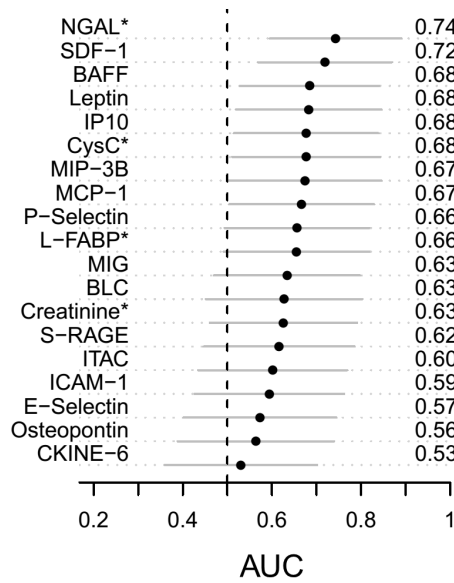

24h – value: CKD

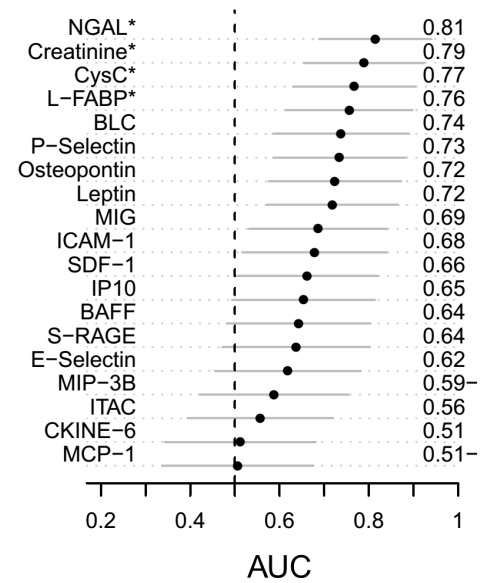

Pre-OP – value: non-CKD

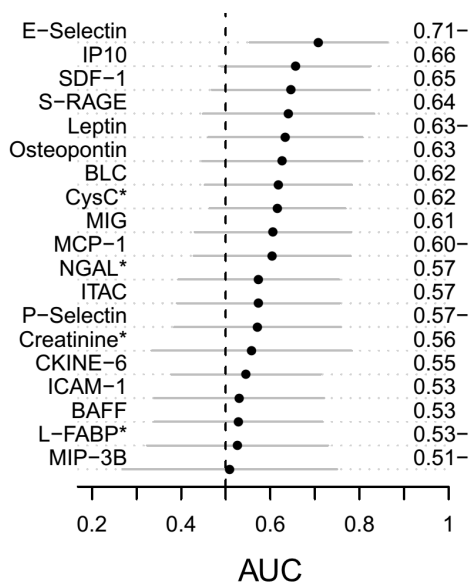

4h – value: non-CKD

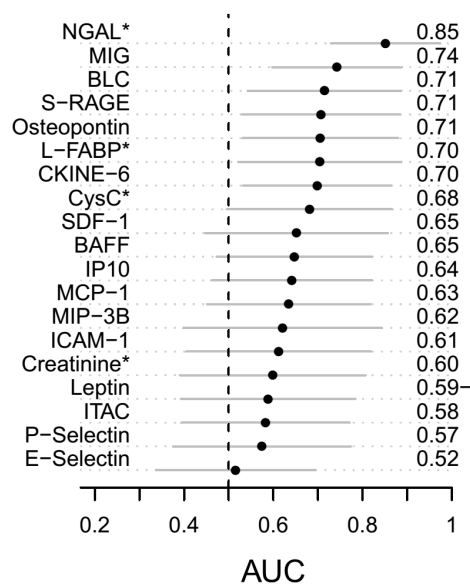

24h – value: non-CKD

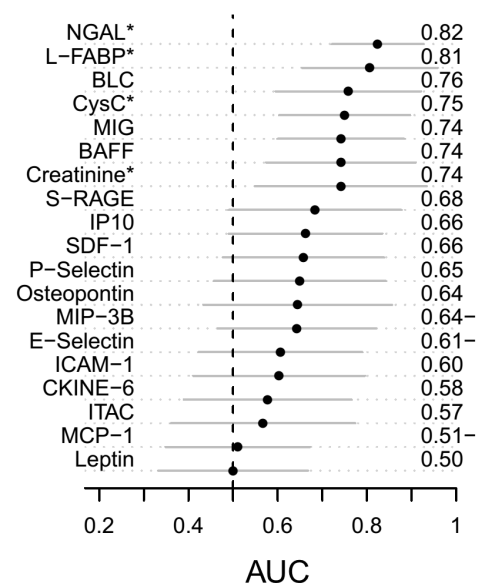

Supplement: S3 Fig — Ranking of biomarkers in plasma according to their preoperative (pre-OP) and postoperative (4h and 24h after CPB) AUC-ROC performance for risk stratification and early diagnosis of AKI stratified in CKD and non-CKD subgroups. AUC values <0.5 were expressed as 1-AUC indicated by AUC−. Parameters marked with * were measured with the assays indicated in the methods section, all others were measured using the Human CustomMAP. The confidence intervals for AUCs were calculated with the DeLong method. Abbreviations: BAFF, B-cell activating factor; BLC, B lymphocyte chemoattractant, chemokine C-X-C motif ligand (CXCL) 13; CKINE-6, chemokine with 6 cysteines, chemokine C-C motif ligand (CCL) 21; CysC, cystatin C; ICAM-1, intercellular adhesion molecule 1, cluster of differentiation (CD) 54; IP10, interferon-γ-induced protein 10, CXCL 10; ITAC, interferon-inducible T-cell alpha chemoattractant, CXCL11, IP9; L-FABP, liver-type fatty acid-binding protein; MCP-1, monocyte chemotactic protein-1, CCL2; MIG, monokine induced by interferon-γ, CXCL9; MIP3B, macrophage inflammatory protein-3ß, CCL 19; NGAL, neutrophil gelatinase-associated lipocalin; SDF-1, stromal cell-derived factor-1, CXCL12; S-RAGE, soluble receptor for advanced glycosylation end products. (PDF) [file pone.0145042.s003.pdf]

Pre-OP - value: CKD

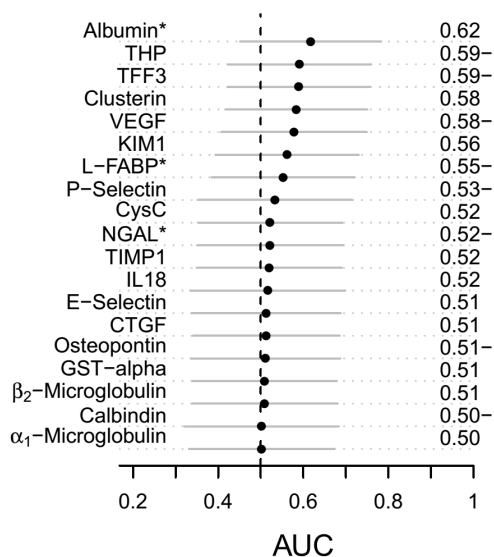

4h - value: CKD

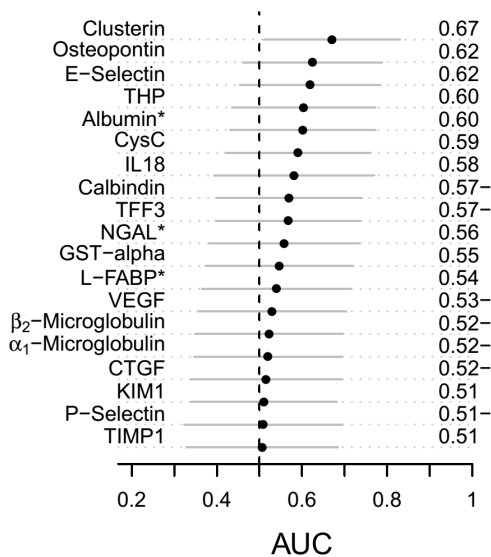

24h - value: CKD

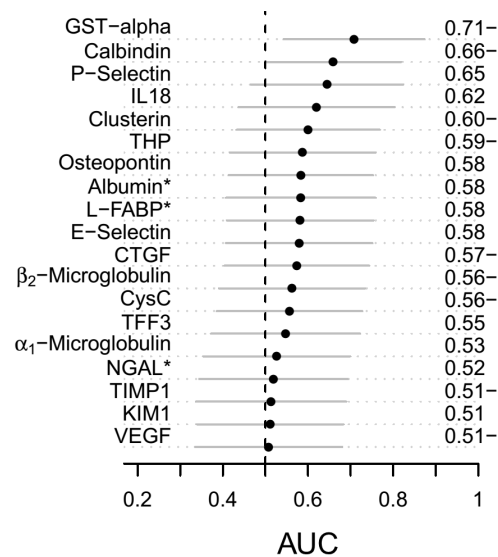

Pre-OP - value: non-CKD

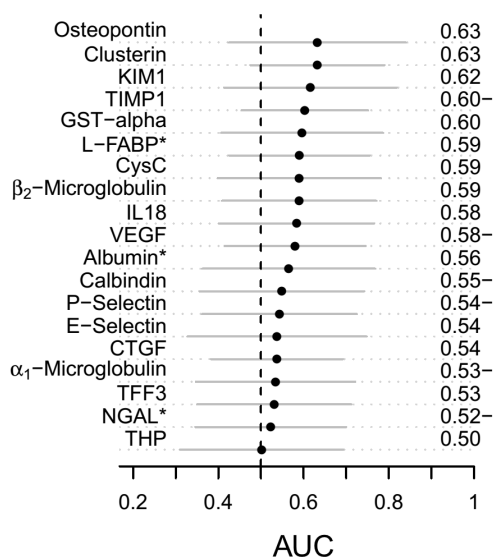

4h - value: non-CKD

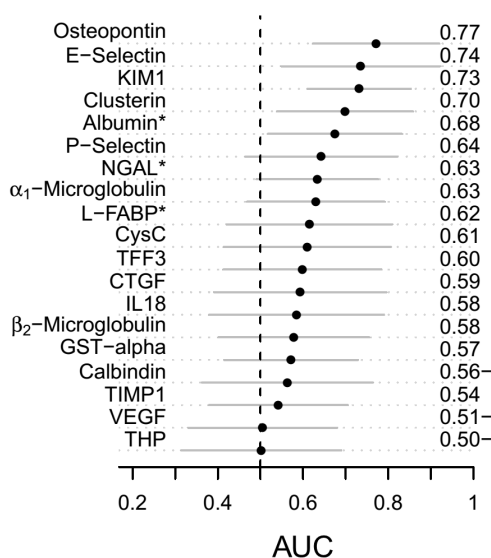

24h - value: non-CKD

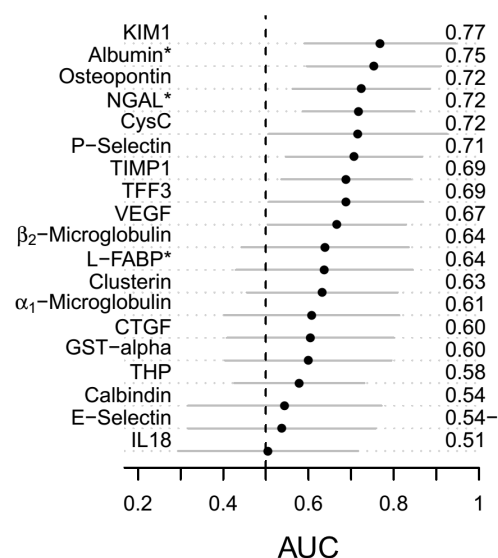

Supplement: S4 Fig — Ranking of biomarkers in urine according to their preoperative (pre-OP) and postoperative (4h and 24h after CPB) AUC-ROC performance for risk stratification and early diagnosis of AKI stratified in CKD and non-CKD subgroups. AUC values <0.5 were expressed as 1-AUC indicated by AUC−. Parameters marked with * were not included in the Human KidneyMAP® and were measured separately. The confidence intervals for AUCs were calculated with the DeLong method. Abbreviations: CTGF, connective tissue growth factor; CysC, Cystatin C; GSTα, glutathione S-transferase-α; IL18, interleukin 18; KIM1, kidney injury molecule 1; L-FABP, liver-type fatty acid-binding protein; NGAL neutrophil gelatinase-associated lipocalin; THP, Tamm-Horsfall protein; TIMP1, tissue inhibitor of metalloproteinases 1; TFF3, trefoil factor 3; VEGF, vascular endothelial growth factor. (PDF) [file pone.0145042.s004.pdf]

# Urinary NGAL vs plasma NGAL

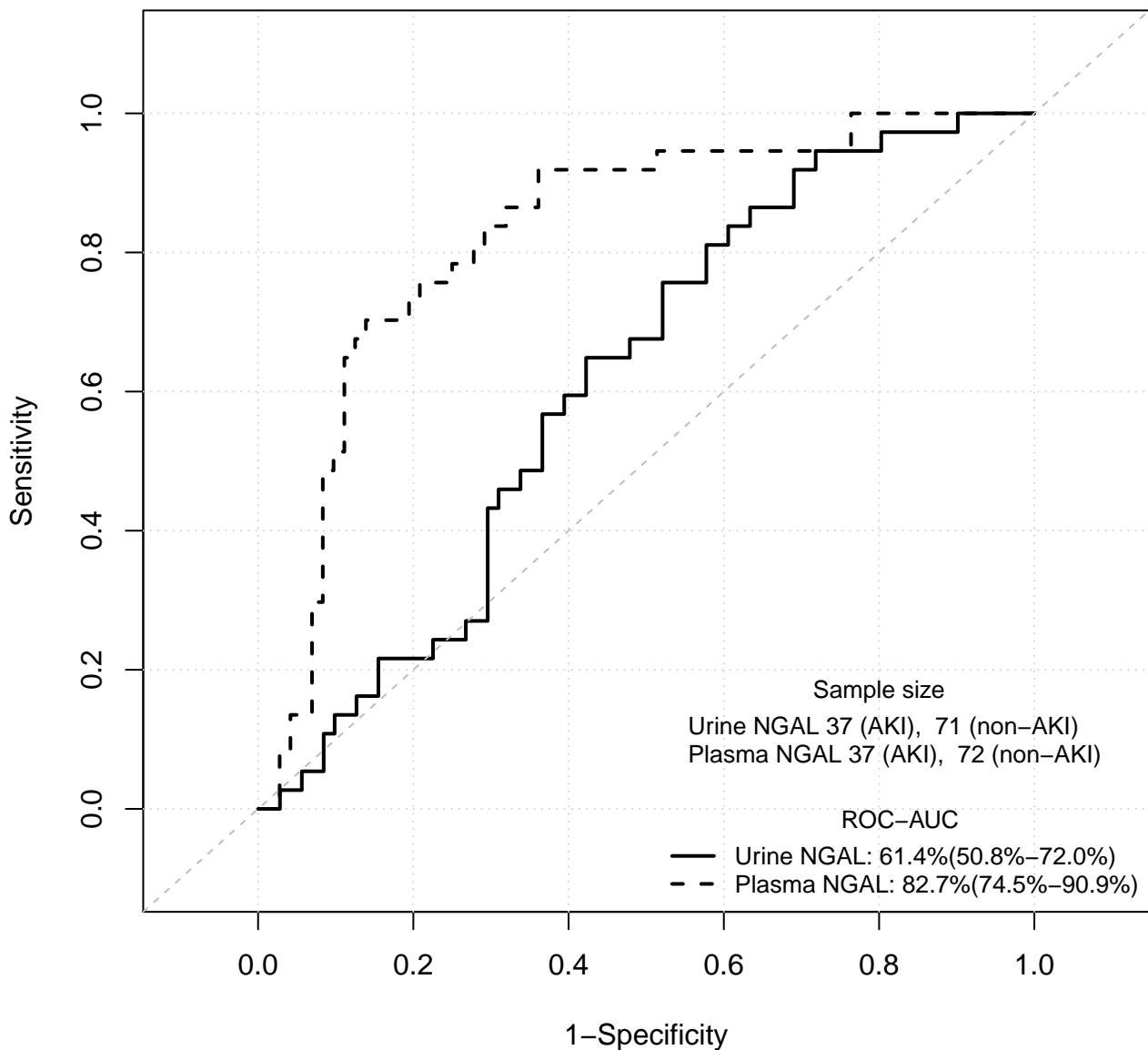

Supplement: S5 Fig — AUC values are given as mean (95% confidence intervals). Urinary NGAL is normalized to urinary creatinine. (PDF) [file pone.0145042.s005.pdf]
