# Supplementary material for: Comparison of Plasma and Urine Biomarker Performance in Acute Kidney Injury
Source: PLoS One. 2015 Dec 15;10(12):e0145042. doi: 10.1371/journal.pone.0145042 (PMC4682932; doi:10.1371/journal.pone.0145042)
Supplement: S1 Table — For categorical variables the first column (“All”) shows column percentages. The columns “non-AKI” and “AKI” contain row percentages. Abbreviations: ACE, angiotensin converting enzyme; ARB, angiotensin receptor blocker; ICU, intensive care unit; IMC, intermediate care unit; max, maximum; min, minimum. (PDF) [file pone.0145042.s006.pdf]

S1 Table: Additional characteristics of study participants stratified by AKI

		All (n=110)	Non-AKI (n=73)	AKI 1-3 (n=37)	P
**Past medical history**					
Diabetes mellitus, n (%)	no	66 (60.0)	47 (71.2)	19 (28.8)	0.21
	yes	44 (40.0)	26 (59.1)	18 (40.9)	
Arterial Hypertension, n (%)	no	2 (1.8)	2 (100.0)	0 (0.0)	0.54
	yes	108 (98.2)	71 (65.7)	37 (34.3)	
Congestive heart failure, n (%)	no	20 (18.2)	15 (75)	5 (25)	0.44
	yes	90 (81.8)	58 (64.4)	32 (35.6)	
Angina pectoris, n (%)	no	33 (30.0)	21 (63.6)	12 (36.4)	0.53
	stable	28 (25.5)	21 (75.0)	7 (25.0)	
	unstable	49 (44.5)	31 (63.3)	18 (36.7)	
Hypercholesterolemia, n (%)	no	9 (8.2)	5 (55.6)	4 (44.4)	0.48
	yes	101 (91.8)	68 (67.3)	33 (32.7)	
Peripheral arterial disease, n (%)	no	94 (85.5)	63 (67.0)	31 (33.0)	0.77
	yes	16 (14.5)	10 (62.5)	6 (37.5)	
Nicotine abuse, n (%)	no	94 (85.5)	61 (64.9)	33 (35.1)	0.57
	yes	16 (14.5)	12 (75.0)	4 (25.0)	
Cerebrovascular disease, n (%)	no	70 (63.6)	49 (70.0)	21 (30.0)	0.30
	yes	40 (36.4)	24 (60.0)	16 (40.0)	
Chronic obstructive pulmonary disease, n (%)	no	90 (81.8)	60 (66.7)	30 (33.3)	1.00
	yes	20 (18.2)	13 (65.0)	7 (35.0)	
Liver disease, n (%)	no	107 (97.3)	70 (65.4)	37 (34.6)	0.54
	yes	3 (2.7)	3 (100.0)	0 (0.0)	
**Preoperative medication**					
β-blockers, n (%)	no	27 (24.5)	19 (70.4)	8 (29.6)	0.64
	yes	83 (75.5)	54 (65.1)	29 (34.9)	
ACE inhibitors or ARBs, n (%)	no	23 (20.9)	16 (69.6)	7 (30.4)	0.80
	yes	87 (79.1)	57 (65.5)	30 (34.5)	
Statins, n (%)	no	28 (25.5)	19 (67.9)	9 (32.1)	1.00
	yes	82 (74.5)	54 (65.9)	28 (34.1)	
Diuretics, n (%)	no	43 (39.1)	30 (69.8)	13 (30.2)	0.67
	yes	67 (60.9)	43 (64.2)	24 (35.8)	
**Outcome**					
Length of hospital stay (days), median (min-max)		14 (3-78)	13 (3-78)	16 (7-72)	*0.05*
Length of stay on ICU (days), median (min-max)		1 (0-55)	1 (0-3)	2 (0-55)	*<0.001*
Length of stay on ICU+IMC (days), median (min-max)		4 (0-63)	3 (2-18)	6 (0-63)	*<0.001*

For categorical variables the first column (“All”) shows column percentages. The columns “non-AKI” and “AKI” contain row percentages. Abbreviations: ACE, angiotensin converting enzyme; ARB, angiotensin receptor blocker; ICU, intensive care unit; IMC, intermediate care unit; max, maximum; min, minimum.
