# Supplementary material for: Comparison of Plasma and Urine Biomarker Performance in Acute Kidney Injury
Source: PLoS One. 2015 Dec 15;10(12):e0145042. doi: 10.1371/journal.pone.0145042 (PMC4682932; doi:10.1371/journal.pone.0145042)
Supplement: S2 Table — Sensitivities (given as point estimates and 95% confidence intervals) and cutoff values of plasma and urinary biomarkers were determined from the AUC curves in Figs 2 and 3 setting the specificity to 75%. Biomarkers in urine are normalized to urinary creatinine (Crea). (PDF) [file pone.0145042.s007.pdf]

S2 Table: Test characteristics of plasma and urinary AKI biomarkers

Biomarker	Time point	AUC	p	Sensitivity	Cutoff
pCystatin C (mg/L)	preop	0.71	<0.001	0.51 (0.32,0.73)	1.39
	2h	0.72	<0.001	0.54 (0.35,0.73)	1.27
	4h	0.76	<0.001	0.70 (0.54,0.84)	1.15
	24h	0.80	<0.001	0.70 (0.49,0.86)	1.35
pNGAL (µg/L)	preop	0.67	<0.005	0.54 (0.35,0.70)	93.87
	2h	0.81	<0.001	0.76 (0.54,0.89)	173.43
	4h	0.83	<0.001	0.78 (0.62,0.95)	178.20
	24h	0.84	<0.001	0.86 (0.46,0.97)	137.61
pL-FABP (µg/L)	preop	0.56	0.35	0.31 (0.17,0.50)	7.06
	2h	0.72	<0.001	0.55 (0.32,0.78)	12.86
	4h	0.73	<0.001	0.51 (0.30,0.78)	12.32
	24h	0.82	<0.001	0.76 (0.54,0.92)	5.15
uACR (mg/g Crea)	preop	0.61	0.06	0.38 (0.22,0.59)	32.56
	2h	0.63	<0.05	0.42 (0.17,0.64)	58.18
	4h	0.65	<0.01	0.51 (0.27,0.68)	34.80
	24h	0.66	<0.01	0.57 (0.40,0.74)	30.08
uKIM1 (µg/g Crea)	preop	0.58	0.15	0.38 (0.19,0.59)	0.72
	2h	0.61	0.06	0.51 (0.22,0.68)	1.25
	4h	0.56	0.29	0.30 (0.14,0.54)	2.04
	24h	0.61	0.06	0.47 (0.28,0.64)	4.57
uNGAL (µg/g Crea)	preop	0.50	0.99	0.19 (0.05,0.43)	34.46
	2h	0.57	0.25	0.27 (0.14,0.46)	83.26
	4h	0.61	0.05	0.24 (0.11,0.57)	60.58
	24h	0.65	<0.05	0.33 (0.14,0.67)	56.86
uL-FABP (µg/g Crea)	preop	0.56	0.28	0.30 (0.14,0.51)	2.24
	2h	0.57	0.22	0.32 (0.19,0.49)	24.44
	4h	0.60	0.08	0.38 (0.19,0.57)	10.86
	24h	0.65	<0.01	0.53 (0.36,0.69)	10.72

Sensitivities (given as mean and 95% confidence intervals) and cutoff values of plasma and urinary biomarkers were determined from the AUC curves in **Fig 2** and **Fig 3** setting the specificity to 75%. Biomarkers in urine are normalized to urinary creatinine (Crea).
