# Supplementary material for: Comparison of Plasma and Urine Biomarker Performance in Acute Kidney Injury
Source: PLoS One. 2015 Dec 15;10(12):e0145042. doi: 10.1371/journal.pone.0145042 (PMC4682932; doi:10.1371/journal.pone.0145042)
Supplement: S3 Table — The relationship between plasma creatinine and various (A) plasma and (B) urinary AKI biomarkers at indicated time points was analyzed by Spearman’s rank correlation coefficient. All biomarkers in urine are normalized to urinary creatinine. (PDF) [file pone.0145042.s008.pdf]

S3 Table: Correlation of plasma creatinine with plasma and urinary AKI biomarkers

(A) Plasma AKI biomarkers

Biomarker	preop	4h	24h
Creatinine	1.00	1.00	1.00
NGAL	0.54	0.58	0.68
Cystatin C	0.54	0.70	0.70
MIG	0.42	0.31	0.50
Osteopontin	0.28	0.21	0.44
S-RAGE	0.24	0.30	0.50
L-FABP	0.21	0.24	0.49
IP10	0.21	0.15	0.36
CKINE-6	0.20	0.31	0.26
ICAM-1	0.15	0.23	0.20
SDF-1	0.10	0.20	0.28
MIP-3B	0.07	0.28	0.06
MCP-1	0.07	0.16	0.10
P-Selectin	-0.02	0.08	0.25
BAFF	-0.02	0.09	0.18
ITAC	-0.03	0.13	0.10
Leptin	-0.05	-0.05	0.03
E-Selectin	-0.08	0.09	0.11
BLC	-0.08	0.24	0.23

(B) Urinary AKI biomarkers

Biomarker	preop	4h	24h
TFF3	0.36	0.07	0.07
α1-Microglobulin	0.27	0.15	0.04
Albumin	0.15	0.13	0.08
L-FABP	0.14	0.19	0.12
β2-Microglobulin	0.13	0.11	0.01
E-Selectin	0.10	0.21	0.05
TIMP1	0.07	0.08	-0.01
Clusterin	0.03	0.01	-0.24
Cystatin C	0.00	0.08	-0.07
IL18	-0.02	0.06	0.01
VEGF	-0.04	-0.04	0.03
NGAL	-0.06	0.13	0.20
KIM1	-0.06	-0.11	-0.07
GST-α	-0.10	0.01	-0.30
CTGF	-0.12	-0.05	-0.17
Osteopontin	-0.14	-0.03	-0.07
P-Selectin	-0.18	0.08	0.04
Calbindin	-0.30	-0.28	-0.34
THP	-0.39	-0.06	-0.35

The relationship between plasma creatinine and various (A) plasma and (B) urinary AKI biomarkers at indicated time points was analyzed by Spearman's rank correlation coefficient. All biomarkers in urine are normalized to urinary creatinine.
