# Supplementary material for: Comparison of Plasma and Urine Biomarker Performance in Acute Kidney Injury
Source: PLoS One. 2015 Dec 15;10(12):e0145042. doi: 10.1371/journal.pone.0145042 (PMC4682932; doi:10.1371/journal.pone.0145042)
Supplement: S4 Table — In this study AKI was defined by the AKIN serum creatinine criteria within 72h after surgery. In the TRIBE-AKI study “severe” AKI was defined as AKIN stage ≥2 based on serum creatinine criteria during the entire hospital stay [5,8,65,66]. “Mild” AKI was defined either as RIFLE stage “R” serum creatinine criteria or requirement of acute dialysis [5] or AKIN stage 1 serum creatinine criteria during the entire hospital stay [65,66]. In this study plasma and urine samples were collected 2 or 4h after discontinuation of CPB, whereas in the TRIBE-AKI study first postoperative samples (0- to 6-hour time point) were taken at a median of 0.25 (IQR 0.1 to 0.5) hours after arrival to the ICU. In contrast to the TRIBE-AKI study, in this study urine biomarkers were normalized to urinary creatinine. Data from the TRIBE-AKI study were summarized from [5,8,65,66]. (PDF) [file pone.0145042.s009.pdf]

S4 Table: AUC-ROC analyses for early diagnosis of AKI with renal biomarkers after adult cardiac surgery in comparison to recent studies from the literature

	This study 2h	This study 4h	TRIBE-AKI “mild”	TRIBE-AKI “severe”	Ho et al. [17]
**pNGAL**	0.81	0.83	0.67	0.70	0.71
**pCystatin C**	0.72	0.76			0.69
**pL-FABP**	0.72	0.73			
**uIL-18**	0.69	0.58	0.65	0.74	0.66
**uACR**	0.63	0.65	0.57	0.59	
**uCystatin C**	0.62	0.61	0.67	0.72	0.63
**uKIM1**	0.61	0.56		0.71	0.72
**uNGAL**	0.57	0.61	0.61	0.67	0.72
**uL-FABP**	0.57	0.60		0.61	0.72

In this study AKI was defined by the AKIN serum creatinine criteria within 72h after surgery. In the TRIBE-AKI study “severe” AKI was defined as AKIN stage ≥2 based on serum creatinine criteria during the entire hospital stay [5,8,65,66]. “Mild” AKI was defined either as RIFLE stage “R” serum creatinine criteria or requirement of acute dialysis [5] or AKIN stage 1 serum creatinine criteria during the entire hospital stay [65,66]. In this study plasma and urine samples were collected 2 or 4h after discontinuation of CPB, whereas in the TRIBE-AKI study first postoperative samples (0- to 6-hour time point) were taken at a median of 0.25 (IQR 0.1 to 0.5) hours after arrival to the ICU. In contrast to the TRIBE-AKI study, in this study urine biomarkers were normalized to urinary creatinine. Data from the TRIBE-AKI study were summarized from [5,8,65,66].
